# Cross-Machine Fault Diagnosis with Semi-Supervised Discriminative Adversarial Domain Adaptation

**DOI:** 10.3390/s20133753

**Published:** 2020-07-04

**Authors:** Xiaodong Wang, Feng Liu, Dongdong Zhao

**Affiliations:** Research Center for High-Speed Railway Network Management of Ministry of Education, School of Computer and Information Technology, Beijing Jiaotong University, Beijing 100044, China; wangxiaodong@bjtu.edu.cn (X.W.); ddzhao@bjtu.edu.cn (D.Z.)

**Keywords:** semi-supervised domain adaptation, cross-machine fault diagnosis, discriminability

## Abstract

Bearings are ubiquitous in rotating machinery and bearings in good working conditions are essential for the availability and safety of the machine. Various intelligent fault diagnosis models have been widely studied aiming to prevent system failures. These data-driven fault diagnosis models work well when training data and testing data are from the same distribution, which is not easy to sustain in industry since the working environment of rotating machinery is often subject to change. Recently, the domain adaptation methods for fault diagnosis between different working conditions have been extensively researched, which fully utilize the labeled data from the same machine under different working conditions to address this domain shift diploma. However, for a target machine with seldom occurred faulty data under any working conditions, the domain adaptation approaches between working conditions are not applicable. Hence, the cross-machine fault diagnosis tasks are recently proposed to utilize the labeled data from related but not identical machines. The larger domain shift between machines makes the cross-machine fault diagnosis a more challenging task. The large domain shift may cause the well-trained model on source domain deteriorates on target domain, and the ambiguous samples near the decision boundary are prone to be misclassified. In addition, the sparse faulty samples in target domain make a class-imbalanced scenario. To address the two issues, in this paper we propose a semi-supervised adversarial domain adaptation approach for cross-machine fault diagnosis which incorporates the virtual adversarial training and batch nuclear-norm maximization to make the fault diagnosis robust and discriminative. Experiments of transferring between three bearing datasets show that the proposed method is able to effectively learn a discriminative model given only a labeled faulty sample of each class in target domain. The research provides a feasible approach for knowledge transfer in fault diagnosis scenarios.

## 1. Introduction

Bearings are ubiquitous in rotating machinery and the failure of bearings would increase the downtime and operating cost. Fault diagnosis methods are of great importance to prevent accidents and reduce maintenance cost. These fault diagnosis methods could generally be divided into two categories: model-based methods and data-driven methods. The model-based diagnosis models usually develop dynamic models of the machinery through analytical approaches and finite element analysis. Gupta [1] introduced a 6-DOF (degree of freedom) model of a bearing and Adams [2] developed a 29-DOF model for a shaft supported by two rolling element bearings. Finite element methods have also been investigated between a roller element and a race defect [3]. Data-driven fault diagnosis models employ large amount of monitoring data and historical data to establish the fault diagnosis models without prior physical or expert knowledge. The data-driven fault diagnosis models used the support vector machine (SVM), k-nearest neighbor (KNN), Bayesian approach and artificial neural network (ANN) models. Deep learning approaches provide an automatic feature learning process without manually feature selection in traditional data-driven fault diagnosis models, and have attracted increasing interesting for fault diagnosis research [4]. However, the deep learning methods only work well when enough labeled training data is available, and training and test data are drawn from the same distribution, which is often not applicable in real industrial applications.

In real industrial applications, the working conditions of rotating machines, such as the rotation speeds and loading conditions, are subject to change during operations. The fault diagnosis model trained on one working condition could not generalize well to another working condition while testing, due to the distribution of test data are different from training data. It would be expensive and impractical to collect sufficient faulty data in industrial environments and retrain the models, especially for safety-critical systems. The existence of domain shift and the lack of bearing damages greatly hinder the application of fault diagnosis methods in industrial environments. Thus, the domain adaptation has been proposed to leverage annotated data from one domain, denoted as source domain, to learn the knowledge for another domain, denoted as target domain, with little or even no annotated data. Numerous studies have shown the benefits of knowledge generalization learned from different working conditions of the same machine. The domain discrepancy of feature representations was explicitly reduced by minimizing maximum mean discrepancy (MMD) [5,6], multi-kernel MMD [7,8], CORAL metric [9], and Wasserstein distance [10,11] in bearing fault diagnosis tasks. Considering the quadratic time complexity of MMD [12] and the adversarial learning methods have been extensively explored, the adversarial domain adaptation strategy have also been widely used [13,14].

However, when the target machine has little or even no annotated faulty data under any working condition, the domain adaptation methods between different working conditions are not applicable. Nevertheless, the annotated faulty data may be available in other machines, such as the open datasets or machines from laboratories. It is natural to explore generalizing fault diagnosis models from these different but related machines. Compared with diagnosis tasks between working conditions, the cross-machine diagnosis tasks are basically more challenging. The differences between two domains in mechanical structure of bearings, fault depth, and sampling frequencies lead to larger domain gap than traditional cross-domain fault diagnosis tasks. Less studies on cross-machine fault diagnosis have been carried. In [15] Yang et al. proposed to transfer fault diagnosis model from laboratory bearings to locomotive bearings. Guo et al. [16] proposed a deep convolutional transfer learning network, and the domain-invariant features are learned from adversarial training between feature extractor and domain discriminator, as well as minimizing the MMD distance of features between domains. In [17] Li et al. proposed to use an auto-encoder as a feature extractor, by this means the unlabeled examples are more involved in training, which could perform well given very limited labeled target samples.

When larger domain gap exists and labeled target samples are insufficient, however, learning a domain-invariant model does not necessarily guarantee a target discriminative model. The well-trained model on source domain may deteriorate on target domain due to the large data density of target domain near the decision boundary, and the ambiguous target samples near the decision boundary are prone to be misclassified. To move decision boundaries into lower density regions of target domain, a variety of target discriminative methods have been explored. One approach is conditional-entropy minimization [18] which increases the confidence of classifier output on the unlabeled target data, thus potentially driving the decision boundaries of classifiers to pass through the low-density target regions. Another approach is adding consistency regularization losses computed on unlabeled data. These consistency regularization losses, such as mean teacher [19] and virtual adversarial training [20] measure the discrepancy between predictions of unlabeled data and its perturbations. By minimizing consistency losses, they enforce the low-density separation assumption of semi-supervised learning and implicitly push the decision boundary to pass through low-density regions of unlabeled data. The virtual adversarial training (VAT) and conditional entropy loss are used as combination in Virtual Adversarial Domain Adaptation (VADA) model [21] and co-regularized domain alignment (Co-DA) model [22] for better performance.

Another issue of cross-domain fault diagnosis is the class imbalance problem where the majority of target samples are normal samples and only limited number of faulty samples are available. When class imbalance exists, samples are prone to be classified as the majority category due to higher prior probability the majority category has. To handle the class imbalance, data-level methods, such as oversampling [23] or down-sampling, are proposed to balance the data distributions explicitly, while algorithm-based methods, such as focal loss [24], address the class imbalance by allowing the minority categories to contribute more to the weight updates. However, these methods often require prior knowledge of class distributions.

To cope with the reduction of discriminative and diversity of cross-machine fault diagnosis model, in this paper we employ the semi-supervised domain adaptation approach to fully leverage the sufficient unlabeled data and insufficient labeled data in target domain to improve the model performance. To handle the larger domain shift in cross-machine fault diagnosis tasks, an adversarial domain adaptation method utilizing nuclear norm maximization and variational adversarial training is proposed to achieve prediction discriminability as well as diversity. We also investigate the class imbalance scenario commonly exists in fault diagnosis tasks. Through this approach, only seldom faulty target data is needed to train an efficient fault diagnosis model.

The contributions of this work are:

(1) An intelligent cross-machine fault diagnosis model is proposed. Fully utilizing the labeled data in source domain and unlabeled data in target domain, this model could perform fault diagnosis task effectively while only limited labeled target data is available.
(2) We proved that the batch norm maximization is effective to improve the discriminability decline and diversity decline, which are both caused by the large domain shift.
(3) Experiments between three open dataset of bearing faults are carried to verify the effective of the proposed method, especially under the situation that only 1 or 5 labeled samples from each category are available for target domain.

The remainder of this paper is organized as follows. The background of target discriminative domain adaptation approaches and batch nuclear-norm maximization are discussed in Section 2. The proposed cross-machine fault diagnosis approach is specified in Section 3. Experiments and analysis on semi-supervised domain adaptation tasks and imbalanced semi-supervised domain adaptation tasks are presented in Section 4. We close the paper with conclusions in Section 5.

## 2. Related Works

### 2.1. Cross-Machine Fault Diagnosis Domain Adaptation

Domain shift often occurs when the models trained on one domain, denoted as source domain, and applied the models to another domain, denoted as target domain, which is obstacle to model generalization. Domain adaptation methods aim to align the feature distributions of two domains. Domain-invariant feature representations are usually learned in the shared feature space by minimizing the distribution discrepancy between domains. Tzeng et al. [25] explores the maximum mean discrepancy (MMD) to measure the divergence in high-dimensional shared space between domains. Long et al. proposed to further reduce domain divergence in multiple task-specific layers of deep neural network by utilizing the multi-kernel MMD [26,27]. Correlation alignment (CORAL) [28] minimizes the domain divergence by aligning the second-order statistics of the source and target distributions which is easy to use. DeepCORAL [29] further extends the CORAL to deep network architectures. Center moment discrepancy (CMD) [30] reduces the domain divergence by aligning high order moments across domains.

Inspired by generative adversarial network, many achievements of domain adaptation have been achieved by adversarial training. A domain discriminator is trained to tell which domain the features come from, while the feature extractor is trained to confuse it. The domain discriminator and the feature extractor are trained adversarially until the target features are indistinguishable from the source features. In [31] Ganin et al. proposed a domain adversarial neural network (DANN). DANN introduced a gradient reversal layer that reversed the gradients of domain discriminator. Tzeng et al. [32] proposed an adversarial discriminative domain adaption (ADDA) which provided an uniform view of adversarial domain adaptation methods. Inspired by conditional generative adversarial network (CGAN), Long et al. [33] utilized multilinear conditioning that captures the cross-covariance between feature representations and class predictions named conditional domain adversarial network (CDAN). To make the fault diagnosis models more generalized and robust to varying working conditions of real industrial scenarios, fault diagnosis tasks between different working conditions have been widely studied. Some researchers resort to minimize the domain divergence between different working conditions through maximum mean discrepancy (MMD) [5,6], multi-kernel MMD [7,8] or Wasserstein distance [10,11]. Adversarial based domain adaptation for bearing diagnosis tasks have also been investigated in [13,14,34].

However, the annotated data for a target machine is not always available in industrial applications. The faults may have barely occurred if the target machine is new and works primarily under healthy conditions, so it is difficult to train an accurate fault diagnosis model independently. In this scenario, cross-machine diagnosis tasks are motivated where the source domain is from different but related machines. Compared with the cross-working condition diagnosis, the cross-machine diagnosis is less explored. In a deep convolutional transfer learning network (DCTLN) proposed by Guo et al. [16], domain invariant features between different bearings are learned through adversarial training and MMD distance. In [17] Li et al. proposed to use auto-encoder to project features into shared subspace and MMD distance is employed to minimize the distance between domains. Through theauto-encoder, the sufficient unlabeled target data also contributes to feature learning.

Compared with conventional cross-domain fault diagnosis tasks which transfer from different working conditions of same machine, the cross-machine fault diagnosis tasks may face different mechanical structures, different fault characteristics, and different sampling frequencies between domains. The larger domain gap makes the cross-machine diagnosis tasks commonly more challenging.

### 2.2. Target Discriminative Domain Adaptation Methods

The existing cross-machine fault diagnosis methods focus on learning domain invariant feature distributions between source and target domain. However, learning a domain-invariant model does not necessarily guarantee a target discriminative model, especially when larger domain discrepancy exists and labeled target samples are insufficient. The well-trained models on source domain may deteriorate on target domain due to the large data density near the decision boundary, which violates the cluster assumption of semi-supervised learning. This could lead to annoying ambiguous predictions on target domain.

To move decision boundaries into lower density regions and improve the model performance on target domain, a variety of target discriminative methods have been explored. These methods could be roughly categorized into two categories, namely discrepancy-based methods and conditional entropy-based methods. Saito et al. [35] proposed a maximum classifier discrepancy (MCD) method to align the two domains using decision boundaries of task-specific classifiers. There are two task-specific classifiers in this method, and the discrepancy between predictions of two classifiers measures how far outside the support of the source domain the target samples lie. Then the feature extractor and two classifiers are performed adversarial training where the feature extractor is trained to minimize the discrepancy while the classifiers are trained to maximize the discrepancy on the target samples. The consistency regularization losses, such as a mean teacher [19] and virtual adversarial training [20], measure the discrepancy between predictions made on perturbed unlabeled data points, so as to add consistency regularization losses computed on unlabeled data. By minimizing the consistency losses, they implicitly push the decision boundary away from high-density parts of the unlabeled data.

Conditional-entropy minimization [18] is also a widely used target discriminative method which forces the classifier to be confident on the unlabeled target data, thus potentially driving the classifiers decision boundaries away from the target data. Due to the domain shift, the model prediction on target data is less certain as on source data, resulting in noisy and high entropy outputs. To achieve high prediction certainty, the entropy minimization pushes the examples to nearby examples far from the decision boundary. The entropy could be minimized directly [36,37] or by an adversarial way [38,39]. In [36], Long et al. imposed conditional entropy loss on target domain data, which ensures that the target classifier fits the target-specific structures well.

As pointed by [18], however, without a locally-Lipschitz constraint on the loss function, the decision boundaries can be placed close to the training samples even when the entropy is minimized. VADA and Co-DA both use a combination of virtual adversarial training (VAT) and conditional entropy loss. They are used in combination because VAT without the entropy loss may result in overfitting to the unlabeled data points and the entropy loss without VAT may result in the network not being locally-Lipschitz and thus not resulting in moving the decision boundary away from the data points.

Another issue of entropy minimization, as pointed out by [40,41], is that the entropy minimization may suffer the side effect of prediction diversity reduction. Since the sample numbers of each category in the batch during training may not equal to each other, entropy minimization is prone to push examples near the category boundary into majority categories, even the examples actually belonging to minority categories. It would be even worse if the dataset is class-imbalanced, namely some classes are abundant while other classes are very limited. The class-imbalanced phenomenon naturally exists in many real-world applications as well as the cross-machine bearing fault diagnosis tasks, since the bearings work under healthy status at most of the time. To maintain prediction diversity for minority classes, the imbalance learning methods are widely explored. In [42], under-sampling for majority samples and over-sampling for minority samples using Synthetic minority oversampling technique (SMOTE) method [43] are both conducted to handle the class imbalance issue. GAN is also used for over-sampling purpose to generate samples. Compared with SMOTE approaches which are based on local information, GAN methods learn from the overall class distribution and have better performance than SMOTE [44]. Deep convolutional GAN (DCGAN) [45] and Wasserstein GAN (WGAN) [46] are also explored for imbalanced fault diagnosis tasks. In spite of powerful tools to generate complicated distributions, GANs are prone to mode collapse and are difficult to train. Different from the imbalance learning methods, Wu et al. [41] proposed a diversity maximization method which explicitly maximizes the category diversity of output.

### 2.3. Improving the Target Discriminability and Diversity Through Nuclear-Norm Maximization

An effective and easy-to-implement method to simultaneously improve the target discriminability and diversity of model output is the batch nuclear-norm maximization (BNM). In [40] Cui et al. theoretically find that the discriminability and diversity of domain adaptation could be achieved by nuclear-norm maximization. In this section, we briefly introduce the BNM regularization.

We propose to use Frobenius-norm (F-norm) of prediction output as indication of target discriminability. F-norm has strict opposite monotonicity with the entropy H(A) where A is the prediction output matrix as proved in [40]. Increasing the prediction discriminability could be achieved by maximizing the F-norm.

We measure the feature diversity of domain adaptation by nuclear norm of the features. Assume that two randomly selected vectors, $A_{i}$ and $A_{j}$, should be more linear independent if they belong to different categories. Otherwise, they should be more linear dependent if they belong to same category. Hence rank(A) could be roughly used as an indicator of prediction categories. We approximate the rank function using the nuclear norm $\|A{\|}_{*}$.

As proven in [47], the relationship between $\|A{\|}_{F}$ and $\|A{\|}_{*}$ could be written as

(1) $$\frac{1}{\sqrt{D}}\|A{\|}_{⋆}\leq \|A{\|}_{F}\leq \|A{\|}_{⋆}\leq \sqrt{D}\cdot \|A{\|}_{F}$$

where D=min(B,C), B is batch size, C is the category number of class, $\|A{\|}_{F}$ is the F-norm of output vector A. When increasing the $\|A{\|}_{*}$, the $\|A{\|}_{F}$ could be also enlarged. Hence the feature diversity and discriminability of domain adaptation could be both enhanced by maximizing the nuclear norm $\|A{\|}_{*}$.

To jointly increase the discriminability and diversity of the prediction outputs, we incorporate the batch nuclear-norm maximization (BNM) regularization into model training. Within each batch samples X of length B, the classification output could be denoted as G(X). And the loss function of BNM can be formulated as:

(2) $${ℒ}_{bnm}=-\frac{1}{B}{\left\|G\left(X\right)\right\|}_{⋆}$$

## 3. Proposed Methods

**Figure 1:**
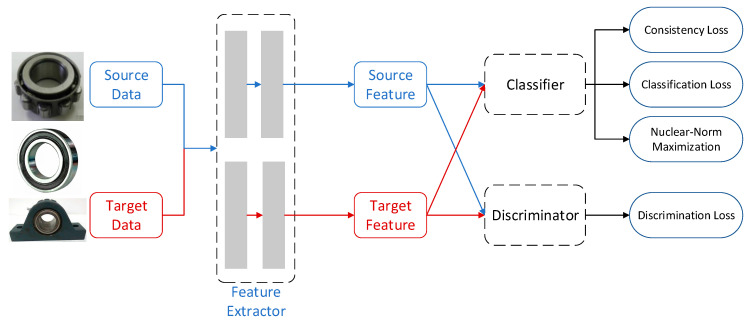
The architecture of adaptation the proposed method.

In this section, we introduce a semi-supervised domain adaptation method for the cross-machine fault diagnosis task. To align the distributions between domains and perform fault diagnosis on target machine with very limited annotated data, the fault diagnosis model learned from related but different machines are generalized. The framework of the proposed method is illustrated in Figure 1. We denote source domain as $D_{s}={\left\{\left(x_{i}^{s},y_{i}^{s}\right)\right\}}_{i=1}^{n_{s}}$, where the samples are fully annotated. $x_{i}^{s}$ represents the sample data and $y_{i}^{s}$ is the corresponding label indicating the health state of bearings. The target domain is denoted as $D_{t}=\left\{D_{t}^{lab},D_{t}^{unl}\right\}$, where limited annotated data $D_{t}^{lab}$ and abundant unannotated data $D_{t}^{unl}$ are available.

The adversarial domain adaptation achieves distribution alignment through adversarial training between feature extractor and domain discriminator. Specifically, the adversarial training involves three components, namely the feature extractor *E*, the label classifier *C,* and the domain discriminator *D*. The feature extractor *E* learns a function $f_{E}(x;\theta_{E})$ parameterized by $\theta_{E}$ that maps the samples x to the feature representation h. Classifier *C* learns a function $f_{C}(f_{E}(x);\theta_{C})$ that maps the feature representation h to class probability output. The domain discriminator *D* learns a function $f_{D}(f_{E}(x);\theta_{D})$ that tells whether the sample comes from the source domain or target domain. The divergence reduction is achieved by adversarial training between feature extractor *E* and domain discriminator *D* where the domain discriminator tries to distinguish which domain the samples come from while the feature extractor tries to fool it.

During training, given access to labeled samples in the source domain and limited labeled samples in the target domain, we first train the feature extractor *E* and classifier *C* on the labeled samples and their corresponding labels in a supervised way, by minimizing the cross entropy loss with K classes:

(3) $${ℒ}_{cls}=-E_{\left(x_{s},y_{s}\right)}\sum_{k=1}^{K}1_{\left[k=y\right]}\log C(E(x_{S}))-E_{\left(x_{t},y_{t}\right)}\sum_{k=1}^{K}1_{\left[k=y\right]}\log C(E(x_{T}))$$

We now train the feature extractor and domain discriminator adversarially. The domain discriminator takes the feature representation and domain label as input, where the samples from source domain is donated as label 0, and the samples from target domain is donated as label 1. The output is the predicted domain label, D(E(x))→{0,1}. The objective function of the discriminator is defined as:

(4) $${ℒ}_{d}=E_{x_{S}}[\log D(E(x_{S}))]+E_{x_{T}}[\log(1-D(E(x_{T})))]$$

To perform domain alignment, the gradient reversal layer proposed in [48] is employed in adversarial training. The overall goal of adversarial training is

(5) $$ℒ={ℒ}_{cls}-{ℒ}_{d}$$

To jointly increase the discriminability and diversity of the prediction outputs, we incorporate the batch nuclear-norm maximization (BNM) regularization into model training, the classification output could be denoted as G(X). The loss function of BNM within each batch samples X of length B can be formulated as:

(6) $${ℒ}_{bnm}=-\frac{1}{B}{\left\|C(E(X))\right\|}_{⋆}$$

To improve the stability of adversarial training and improve the prediction performance, we further impose the local Lipschitz constraint by virtual adversarial training (VAT). VAT perturbs the target data in the most sensitive direction of the model at the input level and minimize the adversarial loss so as to improve the robust of model. The VAT minimization objective is:

(7) $$L_{V}(T)=E_{x_{t}\sim T}\left[\max_{\|r\|\leq \epsilon }D_{KL}\left[h\left(x_{t}\right)\|h\left(x_{t}+r\right)\right]\right]$$

where r represents the virtual adversarial perturbation on input $x_{t}$.

The total loss for the model is defined as:

(8) $$L=L_{C}-\beta L_{D}+\lambda L_{V}+\varphi L_{bnm}$$

where β, λ and φ are hyperparameters.

The algorithm details of the proposed method are presented in Algorithm 1.
**Algorithm 1:** Details of the proposed method**Require**: source data $X^{s}$; target data $X^{t}$; minibatch size B; training step n; 1.Initialize feature extractor, domain critic, classifier with random weights $\theta_{E},\theta_{D},\theta_{C}$2.**for**t=1,…,n do3.Sample labeled minibatch ${\left\{x_{i}^{s},y_{i}^{s}\right\}}_{i=1}^{B}$ from source domain $X^{s}$. Sample unlabeled minibatch ${\left\{x_{i}^{t}\right\}}_{i=1}^{B}$ and labeled minibatch from target domain $X^{T}$.4.calculate the cross-entropy loss: ${ℒ}_{cls}=-E_{\left(x_{s},y_{s}\right)}\sum_{k=1}^{K}1_{\left[k=y\right]}\log C(E(x_{S}))-E_{\left(x_{t},y_{t}\right)}\sum_{k=1}^{K}1_{\left[k=y\right]}\log C(E(x_{T}))$5.calculate the BNM loss:${ℒ}_{bnm}=-\frac{1}{B}{\left\|C(E(X))\right\|}_{⋆}$6.calculate the VAT loss on target samples: $L_{V}(T)=E_{x_{t}\sim T}\left[\max_{\|r\|\leq \epsilon }D_{KL}\left[h\left(x_{t}\right)\|h\left(x_{t}+r\right)\right]\right]$7.calculate the discriminator loss: ${ℒ}_{d}=E_{x_{S}}[\log D(E(x_{S}))]+E_{x_{T}}[\log(1-D(E(x_{T})))]$8.calculate the overall objective: $L=L_{C}-\beta L_{D}+\lambda L_{V}+\varphi L_{bnm}$9.**until**$\theta_{E},\theta_{D},\theta_{C}$ converge

## 4. Experiments and Results

### 4.1. Datasets

The first bearing dataset in use is provided by Case Western Reserve University (CWRU) Bearing Data Center [49]. The vibration signals sampled from the drive-end bearings are used. The faults were manually induced into bearings using electro-discharge machining (EDM). The sampling frequency was 12 kHz. The rotating speed was 1797 rpm.

The IMS bearing data [50] provided by the Center for Intelligent Maintenance Systems, University of Cincinnati, is used as the second dataset. Four Rexnord ZA-2115 double row bearings were performing run-to-failure tests under constant loads. The rotating speed was 2000 rpm and the sampling frequency was 20 kHz. Since the data at different life-cycle stages are considered with different severities, in this study we only adopt the data from the healthy state and severe state.

The third bearing data comes from the centrifugal fan system for rolling bearing fault diagnosis testbed of JiangNan University [51]. The bearings under test is single-row spherical roller bearings. The faults were artificially induced into bearings with a wire-cutting machine. Vibration signals of four categories of bearings including normal, outer-race defect, inner-race defect, and roller element defect. The rotating speed was 1000 rpm and the sampling frequency was 50 kHz.

**Figure 2:**
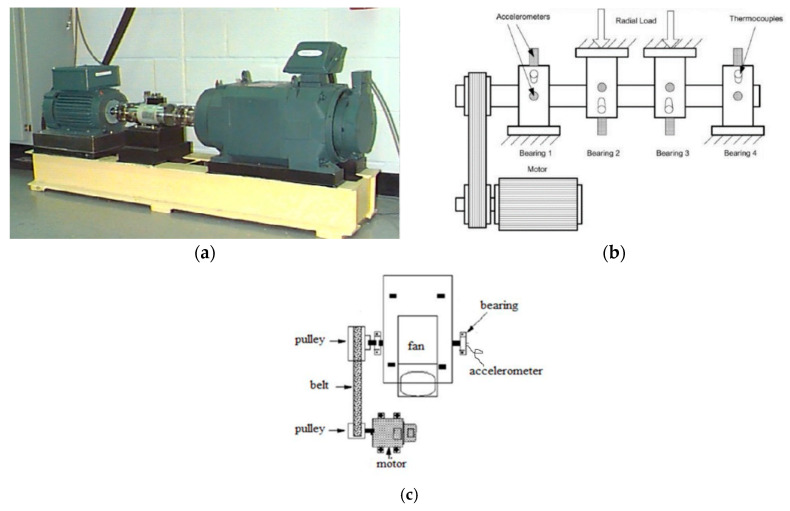
Test rig used in this paper. (**a**) testbed of Case Western Reserve University; (**b**) IMS testbed; (**c**) testbed of JiangNan University.

The three testbeds are shown in Figure 2. The descriptions of bearings of the three datasets are listed for better comparison in Table 1. We could find that the bearings show significant difference between each other, such as the bearing designation, number of roller elements, and sample rate, which makes the cross-machine fault diagnosis tasks more challenging. It should be pointed out that all the datasets share the same label information, namely healthy, inner race fault, outer race fault, and ball fault.

To prepare the training and testing data, we use a sliding window of size 1024 to scan the raw signals and generate the data samples, hence each sample is composed of 1024 sequential points. Each category has 2000 samples, and description of the datasets in use are shown in Table 2.

### 4.2. Implementation Details

The network architecture of proposed method consists of three 1-D convolutional layers, following the rectified linear units (ReLU) activation function and the batch normalization layer. The representation is then flattened and passed to label classifier and domain discriminator. The detailed network architecture is shown in Table 3.

To validate the performance of the proposed method, we compare our method with the following methods. To be fair, the neural network used in our method and the compared methods are kept the same.

- CNN: The model trained on labeled source domain data is used to classify the target samples without domain adaptation.
- Domain adversarial neural network (DANN) proposed by Ganin et al. [48]. Feature distributions are aligned through adversarial training between feature extractor and domain discriminator.
- DCTLN proposed by Guo et al. [16]. Adversarial training and MMD distance are employed to minimize domain shift between domains.
- VADA proposed by Shu et al. [21]. VADA incorporates virtual adversarial training loss and conditional entropy loss to push the decision boundaries away from the empirical data.
- DANN + Entropy Minimization (EntMin). The discriminability of model is improved by entropy minimization on the basis of adversarial training.
- DANN + BNM (BNM). The discriminability of model is further improved by batch nuclear-norm maximization on the basis of adversarial training.

The reported experimental results are averaged by 5 trials to reduce the effect of randomness, and the mean values are provided. All the experiments are implemented using Pytorch and running on Nvidia GTX 2060 GPU.

### 4.3. Case 1: Results and Analysis of Cross-Domain Diagnosis

We evaluate the cross-domain fault diagnosis methods when different numbers of annotated target data are available. The performances on different transfer tasks and different number of labeled target samples are listed. Table 4 shows the cross-machine diagnosis results of different methods. We could find that when no labeled target sample is involved in training, nearly all the methods failed to achieve meaningful diagnosis performance. Higher diagnosis accuracy could be achieved as more labeled target samples are involved in training. When only one target sample is available for each class, the proposed method gains better performance over other methods. When number of target samples raises to 5, nearly all methods could reach best results except tasks using JNU dataset as target. Among different transfer tasks, the transfer tasks of IMS dataset as target domain have higher diagnosis results. This could be explained as that IMS has abundant fault information since the samples of IMS are selected from a run-to-failure experiment.

**Figure 3:**
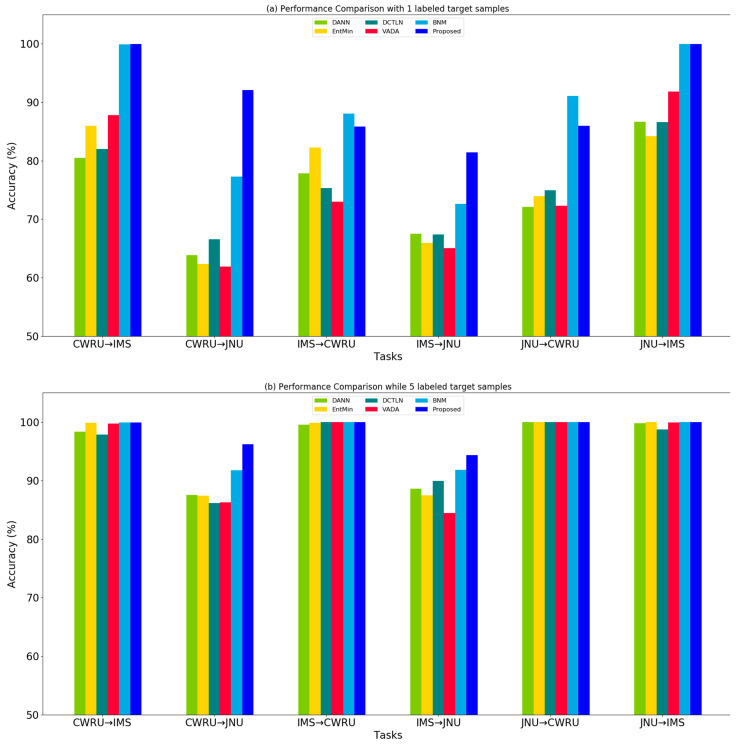
Performance comparison of different methods on different diagnosis tasks when (**a**) number of labeled target samples each class is 1 or (**b**) 5.

To better compare the performances of different methods, we drew the performances of different methods on different tasks when number of target samples is equal to 1 and 5, respectively. When number of labeled target samples is 1, as shown in Figure 3a, the entropy minimization has the average accuracy of 75.77%, which has not shown to effectively improve the performance over DANN with average accuracy of 74.73% except the tasks between IMS and CWRU. By contrast, the proposed method has the average accuracy of 97.86% and achieves highly precision nearly on all tasks. When number of labeled target samples is 5, as shown in Figure 3b, the tasks except CWRU → JNU and IMS → JNU are almost perfectly classified.

**Figure 4:**
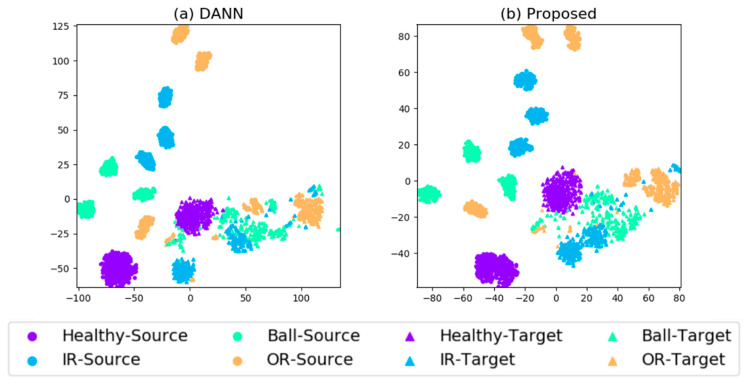
t-SNE results of task ‘CWRU→JNU’ through method (**a**) DANN, (**b**) proposed method. The number of target samples each class is one. Both the features of source domain and target domain are drawn into single images for better visualization. Two shapes represent two domains (square for source domain and circle for target domain), and four colors represent four classes.

We draw the representations of DANN and proposed method via t-SNE for comparison in Figure 4. The task ‘CWRU → IMS’ with number of labeled target sample of 1 is shown. The target features with category ‘IR’ and ‘OR’ are partially overlapped in DANN, and they are better separated in our proposed method. By imposing the target discriminative regularization, the ambiguous samples near the decision boundaries are better separated.

### 4.4. Case 2: Cross-Domain Diagnosis under Class-Imbalanced Scenarios

To further explore the cross-domain diagnosis tasks under class-imbalanced scenarios, we explicitly reduce the unlabeled faulty samples in target domain. Three imbalanced scenarios are composed with different imbalance rate, as shown in Table 5. Firstly, the target dataset is equally split as training set and testing set. Then, the faulty classes in training set are reduced to different extent. For fair comparison, the number of labeled target samples each class is fixed to 5. The results of the experiments are shown in Table 6.

From the results we could find that, the imbalanced scenarios have negligible effect on tasks where CWRU and IMS are target domains. For tasks where JNU is the target domain, the proposed method has obvious improvement over compared methods.

### 4.5. Case 3: Experiments with Environmental Noise

Environmental noise commonly exists in industrial environment, and the vibration signal will be inevitably affected by the noise, which may deteriorate the performance of the fault diagnosis methods. Since the target machine may suffer a different level of noise disturbance, in this section, we evaluate the fault diagnosis performance under environmental noise. The testing vibration signal is added with the additive white Gaussian noise of different signal-to-noise ratio (SNR) which is defined as:

(9) $${\mathrm{SNR}}_{\mathrm{dB}}=10\log_{10}\left(\frac{P_{\mathrm{signal}}}{P_{\mathrm{noise}}}\right)$$

**Figure 5:**
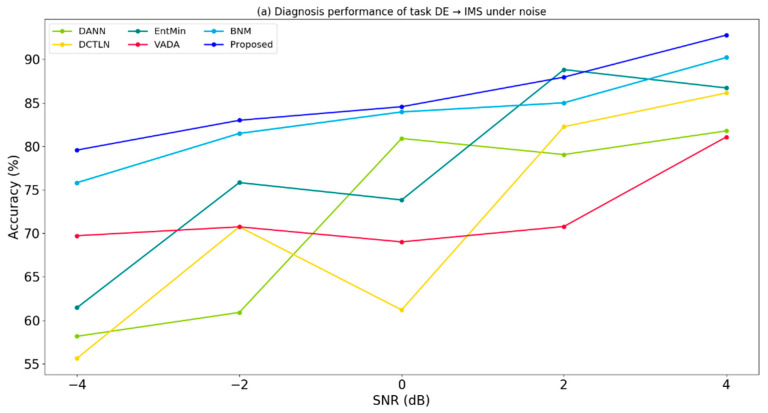

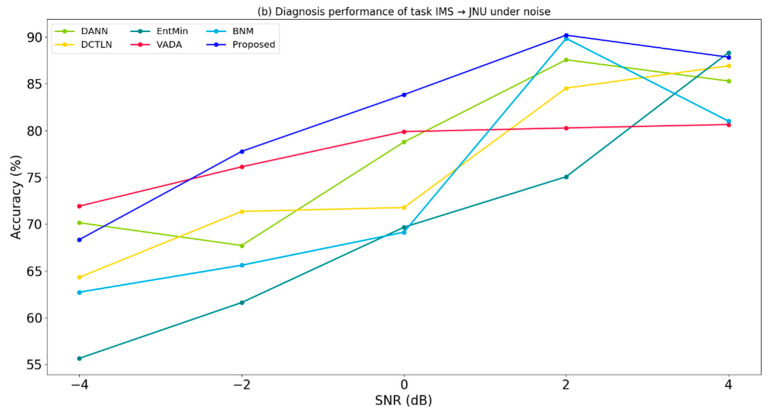
Results of fault diagnosis accuracies under different noise levels in the task (**a**) DE → IMS, (**b**) IMS → JNU. The number of target samples each class is 5.

We test the different diagnosis models under noise level from −4 dB to 4 dB. The result of task ‘DE -> IMS’ is shown in Figure 5a and task ‘IMS -> JNU’ is shown in Figure 5b. The number of labeled target samples each class is fixed to 5.

It could be found that the diagnosis accuracies decrease as the signal-to-noise ratio decreases. Among these fault diagnosis models, the proposed method has better performance on both two tasks. This can be explained that the ambiguous samples in our model are better pushed away from the decision boundaries.

## 5. Conclusions

In this paper, we leverage semi-supervised domain adaptation method to address the cross-machine fault diagnosis problem. Cross-machine fault diagnosis is a promising method to address the dilemma of labeled data insufficiency in data-driven fault diagnosis tasks, especially when labeled data from different working conditions of same machine are unavailable. While it provides the opportunity to generalize the fault diagnosis knowledge from different machines, the cross-machine fault diagnosis is generally more challenging, since the large domain gap between machines make the ambiguous samples near decision boundary are prone to be misclassified, and the data imbalance scenario in target domain makes the faulty samples are prone to be misclassified as normal healthy category. In our method, feature alignment is achieved by adversarial domain adaptation, then the ambiguous samples near decision boundary are further reduced by batch nuclear-norm maximization and virtual adversarial training. Experiments on three different bearing diagnosis scenarios verify the efficacy of our proposed approach when only one labeled target samples each class are available. The class imbalance scenarios also are investigated.

Although the proposed method is effective for cross-machine fault diagnosis, some limitations exist when applying this method. The semi-supervised method assumes there are a few labeled samples in target machine. In our method, adding one labeled sample to each class of target dataset will dramatically increase the accuracy of model prediction. However, in real applications it may be difficult to obtain these labeled data since it is sometimes expensive or infeasible to perform the experiment. In this case, generating labeled data using simulation or digital twin may be a feasible method. Through the virtual experiments, the simulation data could be easily collected, and the annotation information is also available, but with the cost of larger domain gap between simulation data and sensory data. In the future, we plan to explorer the domain adaptation tasks between simulation signals and real signals.

## Figures and Tables

**Table 1:** Characteristics of bearings in three datasets.

Dataset	Bearing in Use	Bearing Category	# of Rollers	Fault Type	Rotating Speed (rpm)	Sample Rate (Hz)
CWRU	6205-2RS JEM SKF	deep groove ball bearing	9	induced using electro-discharge machining	1797	12k
IMS	Rexnord ZA-2115	double-row spherical roller bearing	16	test-to-failure experiments	2000	20k
JNU	N/A	single-row spherical roller bearing	13	induced using wire-cutting machine	1000	50k

**Table 2:** Details of the dataset used in this experiment.

Dataset	Class Label	1	2	3	4
CWRU	Fault Location	Inner	Outer	Ball	Healthy
	Fault depth	14	14	14	14
IMS	Fault Location	Inner	Outer	Ball	Healthy
	Fault depth	Serv.	Serv.	Serv.	Serv.
JNU	Fault Location	Inner	Outer	Ball	Healthy
	Fault depth	N/A	N/A	N/A	N/A

**Table 3:** Details of the networks used in this experiment.

Component	Layer Type	Kernel	Stride	Channel	Activation
Feature Extractor	Convolution1	32 × 1	2 × 1	8	Relu
	BN1				
	Convolution2	16 × 1	2 × 1	16	Relu
	BN2				
	Convolution3	8 × 1	2 × 1	32	Relu
	BN3				
Label Classifier	Fully connected 1	500		1	Relu
	Fully connected 2	4		1	Relu
Domain Discriminator	Fully connected 1	500		1	Relu
	Fully connected 2	2		1	Relu

**Table 4:** Results of diagnosis accuracy under different number of labeled target samples.

Tasks	Number	Accuracy (%)						
		CNN	DANN	DCTLN	EntMin	VADA	BNM	Proposed
CWRU → IMS	0	41.94	40.52	25.62	25.73	27.45	35.62	50.62
	1	/	80.47	81.98	85.95	87.76	99.90	99.95
	5	/	98.33	97.81	99.84	99.74	99.89	99.91
	10	/	99.25	99.01	99.47	99.86	99.96	99.98
CWRU → JNU	0	23.79	24.64	24.79	25.01	25.57	25.05	31.20
	1	/	63.82	66.56	62.36	61.89	77.26	92.07
	5	/	87.55	86.20	87.40	86.32	91.79	96.18
	10	/	93.09	95.16	96.35	93.18	95.57	97.24
IMS → CWRU	0	41.03	49.11	49.32	50.21	50.16	30.68	26.04
	1	/	77.85	75.31	82.26	72.99	86.04	85.85
	5	/	99.56	100.00	99.86	100.00	100.00	100.00
	10	/	99.98	100.00	100.00	100.00	100.00	100.00
IMS → JNU	0	25.81	25.83	26.46	25.36	25.83	27.81	28.75
	1	/	67.48	67.40	65.95	65.07	72.61	81.41
	5	/	88.64	89.95	87.50	84.44	91.84	94.38
	10	/	94.95	95.00	93.91	94.25	94.65	96.51
JNU → CWRU	0	35.64	25.26	25.10	24.95	25.68	24.27	24.95
	1	/	72.09	74.95	73.92	72.31	89.29	87.94
	5	/	100.00	100.00	99.98	99.98	100.00	100.00
	10	/	100.00	100.00	99.98	99.98	100.00	100.00
JNU → IMS	0	40.52	41.72	41.41	44.22	27.03	55.99	49.01
	1	/	86.67	86.56	84.19	91.79	99.95	99.97
	5	/	99.79	98.70	99.97	99.93	100.00	100.00
	10	/	99.29	100.00	99.78	99.74	99.97	100.00

**Table 5:** Details of the imbalanced target dataset used in case 2 of this experiment.

Scenarios	Number of Unlabeled Target Samples				
	Healthy	IR	Ball	OR	Test
**#1**	50%	25%	25%	25%	50%
**#2**	50%	10%	10%	10%	50%
**#3**	50%	5%	5%	5%	50%

**Table 6:** Results of diagnosis accuracy under imbalance scenarios. The number of labeled target samples each class is fixed to 5.

Tasks	Imbalanced Scenarios	Accuracy					
		DANN	DCTLN	EntMin	VADA	BNM	Proposed
CWRU -> IMS	#1	99.37	99.95	99.56	99.59	99.74	99.95
	#2	99.13	99.74	99.06	99.84	99.62	99.84
	#3	99.83	95.73	99.87	99.85	99.98	99.95
CWRU -> JNU	#1	89.46	89.58	88.62	87.50	89.66	95.83
	#2	88.18	83.28	88.15	88.31	88.70	95.19
	#3	90.09	88.02	88.93	87.71	88.78	94.14
IMS -> CWRU	#1	99.77	99.22	99.95	99.92	100.00	100.00
	#2	99.95	100.00	99.98	99.93	100.00	100.00
	#3	99.87	100.00	100.00	100.00	100.00	100.00
IMS -> JNU	#1	89.54	87.97	89.56	84.92	92.38	96.57
	#2	87.58	89.74	87.47	88.75	90.94	96.33
	#3	86.59	86.77	86.38	89.17	90.34	93.39
JNU -> CWRU	#1	99.95	99.95	99.95	100.00	100.00	100.00
	#2	99.95	99.95	100.00	100.00	100.00	100.00
	#3	99.95	99.85	99.93	100.00	100.00	100.00
JNU -> IMS	#1	99.85	99.95	99.87	99.92	99.95	99.93
	#2	99.87	99.69	99.79	99.92	99.98	99.87
	#3	99.95	99.58	99.90	99.95	97.53	99.72
